# Ontology-Based Multiple Choice Question Generation

**DOI:** 10.1155/2014/274949

**Published:** 2014-03-26

**Authors:** Maha Al-Yahya

**Affiliations:** Information Technology Department, College of Computer & Information Sciences, King Saud University, P.O. Box 51178, Riyadh 11543, Saudi Arabia

## Abstract

With recent advancements in Semantic Web technologies, a new trend in MCQ item generation has emerged through the use of ontologies. Ontologies are knowledge representation structures that formally describe entities in a domain and their relationships, thus enabling automated inference and reasoning. Ontology-based MCQ item generation is still in its infancy, but substantial research efforts are being made in the field. However, the applicability of these models for use in an educational setting has not been thoroughly evaluated. In this paper, we present an experimental evaluation of an ontology-based MCQ item generation system known as OntoQue. The evaluation was conducted using two different domain ontologies. The findings of this study show that ontology-based MCQ generation systems produce satisfactory MCQ items to a certain extent. However, the evaluation also revealed a number of shortcomings with current ontology-based MCQ item generation systems with regard to the educational significance of an automatically constructed MCQ item, the knowledge level it addresses, and its language structure. Furthermore, for the task to be successful in producing high-quality MCQ items for learning assessments, this study suggests a novel, holistic view that incorporates *learning content, learning objectives, lexical knowledge,* and *scenarios* into a single cohesive framework.

## 1. Introduction

Ontologies are knowledge representation models that provide a rich platform for developing intelligent applications. Recent advancements in Semantic Web technologies have created an interest among researchers in developing ontology-based applications in numerous research areas. One such research area is the field of question generation (QG), a subfield of artificial intelligence. Recent research has led to the emergence of ontology-based multiple choice question (MCQ) generation. MCQ items have proved to be an efficient tool for measuring the achievement of learners. Instructors could benefit from such systems since the task of manually constructing MCQ items for tests is cumbersome and time-consuming, while it is often difficult to develop high-quality MCQ items. Although ontology-based MCQ generation systems successfully generate MCQ items, little research has evaluated how well these MCQ items are appropriate for use in an educational setting. Such an evaluation is necessary in order to provide guidelines and set requirements for the design and development of ontology-based MCQ generation systems.

This paper aims to address this issue by assessing the performance of these systems in terms of the efficacy of the generated MCQs and their pedagogical value. We present an experimental evaluation of an ontology-based tool for generating MCQ items, the system known as OntoQue [1]. OntoQue is a question generation system that assists the instructor by automatically generating assessment items using domain ontology. The reason why this particular system was chosen is that it was accessible to the researcher, while its generic nature means that it can be used with any domain ontology for any subject.

This paper is organized as follows.  Section 2 presents a background with an overview of ontologies and the task of MCQ generation.  Section 3 provides a review of relevant literature on the task of question generation.  Section 4 presents the OntoQue system and its features, while Section 5 describes the details of the experimental evaluation.  Section 6 presents the results of the evaluation, with Section 7 outlining a set of recommendations and guidelines to consider when designing ontology-based MCQ item generation systems. Finally, Section 8 provides our conclusions and highlights avenues for future research.

## 2. Background on Ontologies

Recent advances in web technologies and the emergence of the Semantic Web have provided a platform for developing intelligent applications, in which ontologies play an important role. Ontologies provide a machine-readable form for describing the semantics of a specific domain. They are knowledge representation structures that describe entities in a domain and their relationships. Entities are the fundamental building blocks of ontologies, and, in turn, they define the vocabulary of an ontology. Classes, object properties, data properties, and individuals are all entities. Classes represent sets of individuals, object and data properties represent relationships in the domain between these individuals, and individuals represent the actual objects in the domain. Using these entities, an ontology facilitates the description of* assertional* knowledge, which provides information about specific individuals, such as class membership. In addition,* terminological* knowledge relates to the classes and relationships that exist and how they relate to one another; an example is subclass and superclass relationships. Terminological knowledge refers to concepts and relations in a domain. For example, a “library” ontology may contain the classes “book” and “journal” and the relations “has-author” and “has-publication-date.” It may also state that “book” and “journal” are types of “publications.” Moreover, the relationships in the ontology may define certain constraints, such as “a book must have at least one author.” With regard to assertional knowledge, the “library” ontology may assert the fact “Book:* A Tale of Two Cities *has-author:* Charles Dickens*.” Ontology entities translated into assertional and terminological knowledge about a domain represent a rich resource from which MCQ items can be automatically generated. They represent asserted facts about a specific domain in a machine-understandable way.  Table 1 shows a number of facts and axioms from the* bibtex *[2] ontology represented in natural language.

The World Wide Web Consortium (W3C), an international organization supporting the development of standards for the Internet, has recommended OWL (web ontology language) as the ontology language for the Semantic Web. OWL ontology contains a collection of statements and expressions; a typical statement is composed of three major elements—subject, predicate, and object—and is thus sometimes referred to as a triple. There are two types of statements in OWL ontologies: facts and axioms. Facts are statements about things in the specific domain. Axioms are statements that describe constraints on entities in the ontology. Algorithm 1 shows the OWL/XML representation for the entry* book* in the* bibtex* ontology [2]. According to OWL ontology, a statement is composed of three major elements,* subject*,* predicate*, and* object*.

## 3. The Question Generation Task

The literature reports on a number of different methodologies used for various purposes in question generation. These methodologies can be classified into syntax-based, template-based, and semantic-based models. The majority of methodologies utilize a natural language resource, from which questions are generated. These resources are either general or domain-specific. The purposes for which questions are automatically generated include assessment [3–5], revision or study questions [6], exercise questions [7], look-back strategy questions [8], problem-solving questions [9], general questions in a specific domain, such as tourism [10], or open-domain questions [11].

In syntax-based approaches, only the syntax of the natural language sentence is considered, not the semantics. The main characteristic of these systems is their heavy reliance on natural language processing (NLP) tools. One of the earliest question generation systems is syntax based [6], with natural language parsers being used to analyze sentence syntax and identify the major components that can be used to form a question. The questions are generated to aid revision and study. However, a major drawback of such an approach is the existence of syntactically ambiguous language sentences, and the only way to parse such sentences correctly is to understand the meaning of the sentence. Another drawback is that the system is language dependent. However, one of the main advantages of this approach is that it is domain independent, so that a natural language sentence in any domain can be used to formulate a question.

Language resources, such as WordNet [12], have been used for the question generation task. Brown et al. [4] describe a system for generating questions for vocabulary assessment using WordNet for MCQ generation. Based on the attributes and lexical relations in WordNet, six types of vocabulary questions are defined: definition, synonym, antonym, hypernym, hyponym, and cloze questions. Natural language text is parsed and tagged with part-of-speech (POS) information. A word is selected from a given text, and then data from WordNet is extracted for all six categories. For example, the definition question requires a definition of the word, which is retrieved from WordNet's gloss. The system selects the first definition that does not include the target word. Although the system exploits the structure of the domain resource (WordNet) for question generation, the questions generated are limited to vocabulary-type questions. Similarly, the system presented in [13] generates cloze questions for assessing the comprehension of a reading text. The system enables the creation, answering, and scoring of text comprehension questions. Cloze questions are generated by parsing a natural language sentence; a random word is deleted and then three random distracters of similar difficulty are chosen from the text.

The system described by Gates [8] uses NLP tools to generate look-back strategy questions. Wh-questions are derived from the natural language text. The text is parsed and analyzed using NLP parsers to generate a parse tree for the declarative sentence. A set of human-defined tree transformation rules and other NLP tools are used to transform the sentence into a wh-question. This system is specifically designed for wh-fact questions only and relies on human-defined rules to transform a sentence into a question.

For other MCQ generation systems based on NLP of textual resources, the similarity measure is obtained by using either language lexicons such as WordNet or computational algorithms that provide a similarity measure between two words [14].

Silveira [15] describes the work-in-progress for developing a general framework for question generation. The input to the system is free text, which is parsed and annotated with metadata. Once annotated, an appropriate question model is selected, and then the question is formulated using natural language.

An example of template-based approaches to question generation is described by Stanescu et al. [16]. The system uses a number of predefined tags and templates, which the instructor can then use to create questions. The text is first displayed in a window, and the instructor then selects a concept and a suitable question template from the set of available templates. The system then parses the selected text and generates questions.

Another example of the template-based approach to question generation, specifically MCQ items, is the work described by [17]. The authors describe task models (templates) that can be used for automatic generations of assessment items. They use Item GeneratOR (IGOR) software [18], which generates models by allowing users to enter text for the stem and to identify variables, constraints, and response choices in order to generate a wide range of MCQ items.

Semantic-based approaches are usually domain dependent. They depend on a semantic model of the domain in order to generate questions. The OntAWare system [19] uses an ontology and generates questions based on knowledge of class-subclass and class-instance relationships. The system provides, among other functionalities for educational content authoring, the semiautomatic generation of learning objects, including questions. The system uses subsumption relationships between classes in order to generate questions, such as “Which of the following items is (or is not) an example of the concept, X?” Although it uses an ontological model of the domain, it does not fully exploit other relationships or constraints in the ontology.

In [10], query templates are analyzed and question patterns predicted. The system uses domain ontology to generate a set of question patterns, which are predicted, with users being asked questions in a specific domain. Natural language text in a specific domain (tourism) was obtained from the Internet and semantically annotated with metadata derived from the ontology to create a triple-based resource description framework (RDF). All triples in the format of 〈class, property, range〉 are generated. By analyzing these triples against a set of randomly selected user queries, two types of questions were identified. The first involves querying the “name” property of a class instance using one or more of its other properties as the constraint(s). The second entails querying a property X, other than the name of a class instance, using its “name” property as the constraint. Although this system utilizes an ontological model, it does not exploit other ontological constructs. It also relies on the analysis of user query patterns in a specific domain.

The system presented by [3] describes the automatic generation of MCQ items from domain ontologies. The semantic relationships between various entities in the ontology are used to assert true/false sentences, which are then used for generating the distracters in question items. The authors describe three main strategies for question generation: class-, property-, and terminology-based strategies.

Similarly, the work described by [20] uses ontologies to generate MCQ items, with the system (SeMCQ) being developed as a plug-in for the protégé ontology editor [21]. Its strategies are similar to those described by [3]. In SeMCQ, all item stems begin with the word “which,” “what,” or “who.” There are two forms for the language and wording of the question stem: “What is a* variable*?” and “Which one of these statements is correct?” The options for a single item are framed in a membership frame, for example “X is a Y.” Items are based on class membership and do not exploit the semantics of the domain, namely, that of object properties. Although this system uses an ontological domain model for question generation, it only focuses on generating the MCQ-type and does not consider other question styles. The system presented by [22] uses OWL ontologies as the source for the domain knowledge and generates tests. The system is based on a number of defined templates for questions from which the system can generate the test items.

For the task of educational assessment, the approach of [23] utilizes ontology not to generate assessment items, but rather to enable students demonstrate their knowledge and understanding of the concepts while creating ontologies. Ontology in this case is used as an assessment item itself and not to generate items.

## 4. Requirements for the MCQ Generation Task

MCQs consist of four major components: the stem or the text stating the question; a set of possible answers called* options*; the* key* or the option that is the correct answer; the incorrect options known as* distracters*. The basic strategy of MCQ item generation is to decide on a suitable* stem*, identify the* key*, and then generate distracters. Distracters should be as close as possible semantically to the key; generating them is considered the most difficult task for instructors.

An ontology-based infrastructure supports the task of stem and distracter generation. Since ontology axioms provide facts about the subject domain, by using these facts, we can enumerate valid statements (RDF triples) about the domain, which form the backbone for generating assessment items. For stem generation, an ontology provides a conceptual model from which the stem's central or major domain concept (key concepts) may be derived. For generating distracters, the ontology structure provides a network graph model that groups concepts within classes and subclasses, which in turn provides a measure of the semantic closeness required when generating distracters. This measure of similarity is derived from human intelligence during the ontological engineering process. Such an approach to the similarity measure provides a basis for computing how close the options are to the key, thus enabling the generation of plausible distracters.

## 5. The OntoQue MCQ Generation System

The OntoQue engine generates a set of assessment items from a given ontological domain. The items include MCQ-type, true/false (T/F), and fill-in (FI) items. The system is implemented using Jena, a Java-based framework API for OWL ontology model manipulation. The OntoQue engine generates items by iterating over all entities (combination of viable statements) in the ontology. Since the aim of this study is to evaluate the quality of MCQ items generated, we will limit our discussion to these types of questions.

### 5.1. Stem Generation

The stem for an MCQ is derived using ontological statements (triples). There are three strategies used by OntoQue for stem generation:* class membership*,* individuals*, and* property*. Class membership provides stems that ask questions of the type “what is the* kind of*.” To generate MCQ stems using this strategy, all defined classes along with their instance members are collected in a group of RDF statements.

For individual-based strategy, all individuals from the domain ontology are listed, and for each individual, we collect all assertions in which the individual is a subject or object. These statements then form the base from which the stem is generated. Subjects and objects are always related through a particular property, and the engine contains algorithms for constructing items from property axioms (characteristics), such as transitive properties.

Statements are extracted and converted to natural language before the subject or object is removed. This removed element will then serve as the key, since the statement is true.

### 5.2. Distracters Generation

In class-based strategies, distracters are randomly generated from a set of classes, excluding the object class. The* key* is the object of the RDF statement. In individual-based strategies, the selected distracters are of the same class as the key. They are generated by randomly selecting three options from a collection of items that are classified as similar to the key option and thus distracting.

## 6. Experimental Evaluation

### 6.1. Data Set

Our study used two domain ontologies for the evaluation: the* HistOnto* and* SemQ* ontologies. The HistOnto ontology was used when developing the OntoQue system, while the SemQ ontology represents Arabic vocabulary (time nouns) using lexical semantic features. The ontological statistics are provided in Table 2.

A preliminary evaluation and filtering was performed by the researcher prior to the experimental evaluation [1]. During this process, only valid MCQ items were filtered to the subsequent stage of our evaluation. This resulted in a total of 235 items for HistOnto and 798 for SemQ. Table 3 shows the details of the MCQ items generated by the system.

### 6.2. Participants and Method

Three instructors with experience in formulating MCQ-type items evaluated the MCQs generated by the system. For each of the two ontologies, they were given a random sample set of 20 MCQ items from the resultant set of questions, resulting in a total of 120 MCQs. Our evaluation strategy was similar to that used by [24]. Evaluators were asked to determine whether each MCQ item did or did not adhere to a set of rules for MCQ design, as shown in Table 4. They were asked to classify whether the MCQ item was worthy or not of being considered as an assessment item. This measure gave an indication of the educational significance of an item. Next, for the worthy items, evaluators were asked to suggest whether the MCQ item required a* minor*, fair, or* major* revision.* Minor* revision describes language proofing of the item.* Fair* revision involves further editing of the MCQ item, such as reordering, inserting, or deleting several words, and replacing one distracter at most.* Major* revision involves substantial rephrasing of the stem and replacing at least two distracters. For* major *revisions, evaluators were asked to describe their reasons if it concerned something other than a violation of the MCQ rule.

### 6.3. Evaluation Metrics

For our evaluation experiment, we used a number of metrics to measure the quality of the MCQ items generated by the system using the HistOnto and SemQ ontologies. Metrics include the* total* number of worthy MCQ items generated, the* modification* measure, and the* quality* of the MCQ item. The modification measure has the following values: “0” for items requiring no revision and “1,” “2,” and “3” for items requiring minor, fair, and major modifications, respectively. The quality measure for each MCQ item corresponds to a numerical value denoting how well the item adheres to the MCQ rules. The quality of an MCQ item is thus the cumulative score of its conformity to the MCQ design rules as shown in Table 4; thus, a high-quality MCQ item would yield a maximum score of 10.

### 6.4. Results

**Figure 1:**
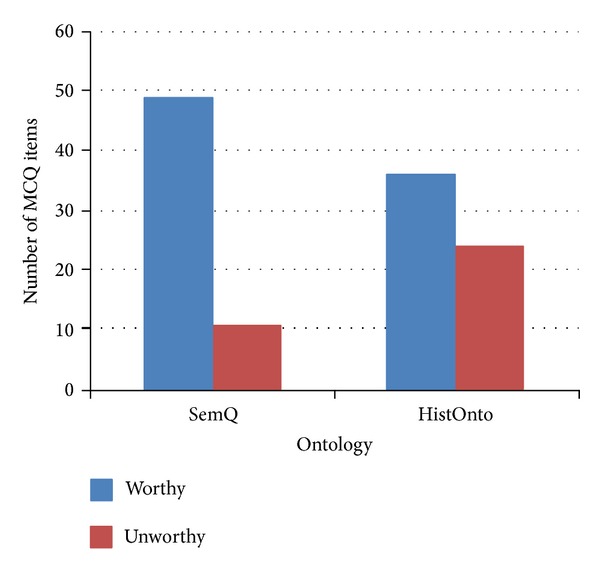
Worthy versus unworthy items.

The evaluation metrics resulting from our experiment are shown in Table 5. These include the number of worthy items, the average quality measure of MCQ items for each ontology, and the modification measure.  Figure 1 shows that for the SemQ ontology, the engine produced better and more educationally significant items than the HistOnto ontology.

**Figure 2:**
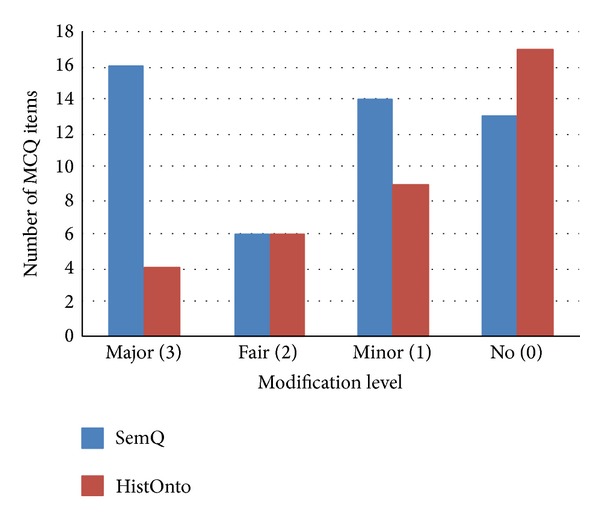
Modification level required.

Figure 2 shows the type of modification required for worthy items for both ontologies, showing that the majority of MCQ items required none or only minor modification: 26 out of 36 items for HistOnt and 27 out of 49 items for SemQ. The MCQ items generated from SemQ required more major and minor modifications than those from HistOnto. Table 6 shows the reasons why evaluators chose to make major modifications to the MCQ items. One of the most popular reasons was the plausibility of the distracters.

**Figure 3:**
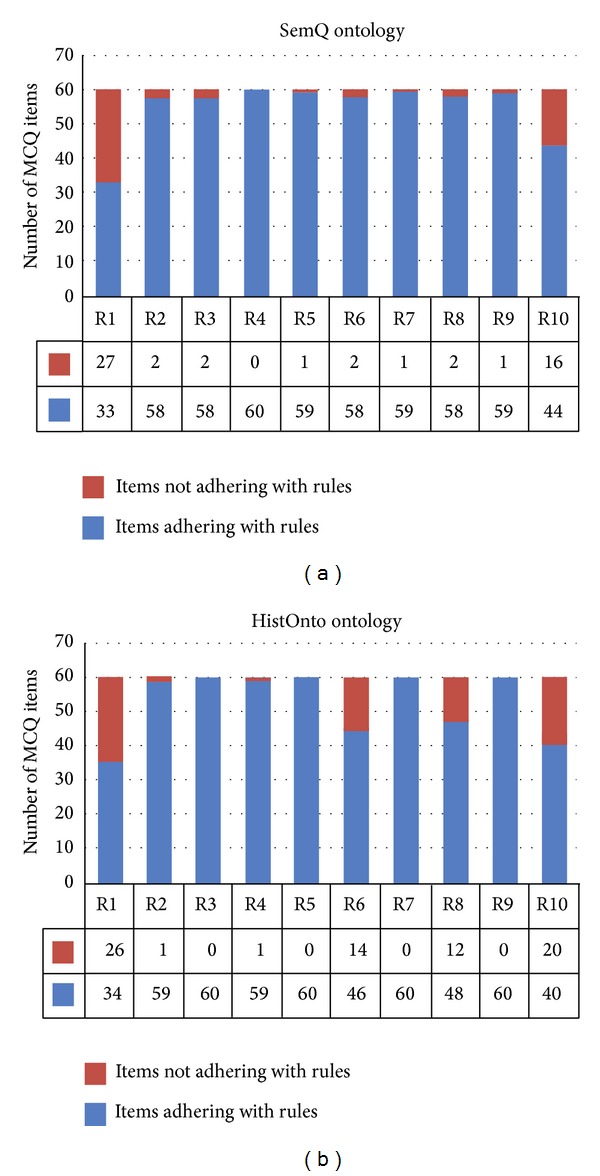
Rule violations details.

With regard to rule violations, the total number of items violating MCQ design rules was similar in both ontologies. For SemQ, 72 rule violations were found compared with 74 for HistOnto. Details of the rule violations are shown in Figures 3(a) and 3(b). From the figure, we observe that, for the SemQ ontology, the majority of rule violations were associated with rules (R1 and R10), while for HistOnto the majority were associated with rules (R1, R6, R8, and R10).

## 7. Discussion

From our experimental evaluation, several important observations may be made. First, with regard to the educational significance of the items generated, the two ontologies differed. Concerning the quality of items generated for both ontologies, the evaluation indicated that the average quality value was acceptable, being 8.8 out of 10, and it did not differ between ontologies. Although the majority of items generated from SemQ were considered to be educationally significant by the evaluators, for the HistOnto ontology, almost half of the items generated were believed to be irrelevant. A possible interpretation can be identified by considering the kind of domain knowledge that each ontology represents. The SemQ ontology represents vocabulary (Arabic words from a specific domain), while the HistOnto ontology describes historical facts. Reexamining the MCQ items that were classified as unworthy, especially in the HistOnto, items dealing with commonsense or general knowledge were identified, although, for the SemQ ontology, this was not the case. Therefore, distracters generated using HistOnto were not plausible. For an ontology-based MCQ generation system to be acceptable, it must provide strategies for distinguishing between commonsense and higher-level knowledge, the latter being what we would prefer to be assessed. There are two possible solutions to this problem: either this knowledge should not be modeled as part of the ontology or it should be annotated with instructional metadata, such as the level of difficulty.

Furthermore, with regard to rule violations, rules R1 (distracter plausibility) and R10 (contains one correct answer) represented the majority of violations in both ontologies. The HistOnto also had a significant number of violations to rules R6 (are grammatically consistent with the stem) and R8 (are all of approximately the same length).

Distracter plausibility is an important aspect of an MCQ item. It is considered one of the major challenges facing instructors when constructing MCQ items. Although the system uses algorithms and techniques to identify options that are similar to the key as distracters, results indicate that this is not sufficient. A plausible distracter makes students think; it is compelling, but at the same time, confusing. One possible approach for creating plausible distracters is to consider students' frequent areas of misunderstanding or their common errors.

Concerning the violations of rule R10 (item contains one correct answer), to overcome this shortcoming, we must verify all options and ensure that these statements are not valid in the ontology; that is, no such assertion exists. However, this may not always be true due to the open world assumption adopted by OWL ontologies.

The issue of the language and grammar in the generated MCQ items is another important issue. The evaluation revealed some violations of rule R6 (options are grammatically consistent with the stem). To make sure that the grammar and syntax of the item are correct, we need to enhance the system with linguistic knowledge about the ontology entities. This can be achieved by using linguistic ontologies, such as LexInfo [25], to associate language-specific metadata with domain ontologies, thus enabling the system to correctly phrase the item.

Finally, as to rule R8 (options are all of approximately the same length), this can easily be implemented in the system by comparing the length of the options.

Examining the reasons why some MCQ items were acceptable but required major modifications, we can see the most common reason was that distracters had to be changed because they were too easy or were incomplete (less than four options). Evaluators also indicated that some of the options were invalid (blank), and some MCQ items did not show a correct answer within the options list but contained more than one correct answer. These are all due to technical issues (program bugs) and can be resolved in future versions of the system.

An important piece of feedback from our evaluators was that the majority of MCQ items concentrated on factual knowledge, thus only addressing the first level of Bloom's taxonomy of cognitive skills required for learning. The evaluators would prefer if the MCQ items addressed a higher level of cognitive skills.

## 8. Recommendations

Ontological constructs (ontology-based systems for MCQ item generation) appear to be an appealing resource for the task of generating MCQ items, since they provide a formal representation of domain knowledge in the form of logical assertions. However, our experimental evaluation revealed a number of shortcomings in such an approach. For systems to produce MCQ items that can be used in real tests, these limitations need to be addressed. Therefore, we suggest the following recommendations for the ontology-based MCQ item generation task.

### 8.1. Educational Significance

Concerning educational significance, we need to address the learning objectives, as the MCQ item measures the level obtained by students for this objective. A learning objective consists of the knowledge required and its (cognitive) level. Neither element was considered when designing the OntoQue system. As a result, the majority of questions were suitable, but not educationally relevant, especially with HistOnto. We must therefore understand the cognitive level addressed by the learning objective in order to provide the necessary information for the system to identify the appropriate keywords to use in the item.

Our evaluation also highlighted the issue of difficulty of a given item, which directly relates to the knowledge level. The system should include measures to identify and control the difficulty level of the generated item. For example, the cognitive level may be an indicator of item difficulty; thus, the higher the level is, the more difficult the item is. Moreover, examining more than one fact may also increase the level of difficulty.

### 8.2. Addressing Higher-Level Thinking

The evaluation showed that the majority of items tested factual knowledge. According to Aiken [26], MCQ items can be used to test higher-level learning skills. To enhance the system so that it can generate MCQ items to measure higher-order thinking, we can employ techniques such as those suggested by Chase and Jacobs [27], which encompass all five levels of cognitive skills. One such method is to use a sequence of MCQ items to test higher-level thinking. Woodford and Bancroft [28] also suggest how MCQ items can be designed to address higher level cognition.

Since the items are essentially derived from knowledge in the ontologies, the type of knowledge and the subject domain are factors that need to be considered when designing MCQ items to measure higher-level cognitive skills. For example, assertion- and reason-based MCQ items are classified as items addressing higher levels. Such items cannot be automatically generated unless the ontology contains causal knowledge (i.e., cause-effect relationships, axioms, and primitives). Currently, OWL does not provide constructs for modeling such knowledge. The ontological engineer may create such relations, but they will only be valid for the local ontology, and these relations must be explicitly specified in the system, thus making it less generic.

Another possibility for devising items that measure higher-level thinking skills is by introducing the element of novelty. Novelty means that the item should assess the objectives in a new context or scenario, which requires students to apply learned knowledge and cognitive skills. Such an approach can be incorporated by providing task templates [17]. Such templates may be designed from scratch by the instructor or derived from the ontology with domain concepts and the aid of an instructor.

### 8.3. Natural Language Generation

One of the major issues identified by evaluators in the MCQ items was the structure of the language used in the stem or options. The generated items would benefit from lexical improvements to the sentence structure and vocabulary. External lexicons, such as WordNet [12], can be used for varying the wording of the item. Moreover, using the grammatical and syntax markup provided by lexical ontologies, such as LMF [29] or LexInfo [25], would enhance the readability and language of the generated item.

### 8.4. Evaluation of MCQ Items

Although our evaluation provided useful results on item quality to guide the ontology-based MCQ item generation task, it did not include results on the application of the generated items in a test. An item will not be high-quality if all learners respond correctly (too easy) or incorrectly (too difficult), because it will not reveal any information about their competences. Since the generated MCQ items were not used in a real test taken by students, the evaluation did not take item analysis into consideration. Item analysis evaluates how well the item functions as a discriminator to the individual competence of each student. Such analysis requires the evaluation of parameters, such as the *P* value defined as the proportion of correct responses and the discrimination index defined as the correlation with the residual scores. Item analysis consists of the following variables [30]: (1) difficulty of the item, (2) its discriminating power, and (3) the usefulness of each distracter. Item analysis indicates the item's difficulty, that is to say, whether it was too easy or hard and how well it discriminated between high and low scorers in the test.

## 9. Conclusion and Future Work

This paper described an experimental evaluation on ontology-based MCQ item generation. The aim of the evaluation was to measure the competence of such system in generating MCQ items usable in educational assessment. The OntoQue system was chosen as the platform for the experiments with two domain ontologies, SemQ (Arabic vocabulary ontology) and HistOnto (history ontology). Results of the evaluation indicate such systems can generate reasonable and acceptable MCQ items, although some limitations were also revealed.

These limitations lay the foundation for future work. Further developments must adopt a holistic view in the MCQ item generation task. This holistic view should incorporate* learning content*,* learning objectives*,* lexical knowledge*, and* scenarios *in a single cohesive framework. We are currently working on such a framework known as “TeGen.” This framework provides a platform for developing MCQ item for tests using ontology-based resources as the standard markup. This framework for test generation combines learning objectives, educational resources (learning content and knowledge), and assessment in a single coherent structure.

## Figures and Tables

**Algorithm 1:**
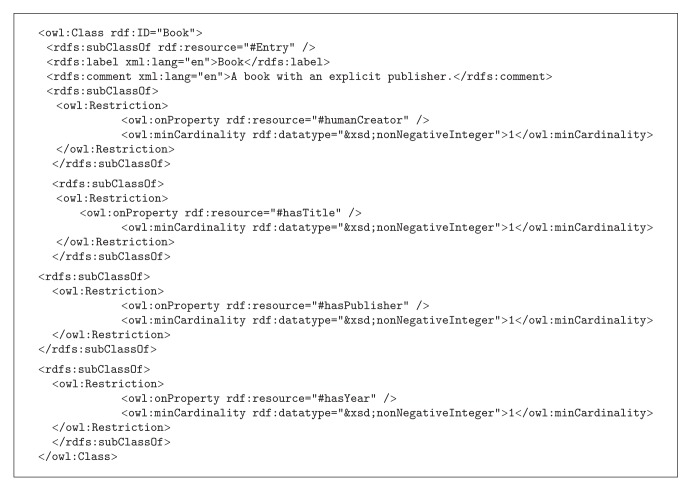
Bibtex OWL/XML ontology for “Book”.

**Table 1:** Sample facts and axioms from the bibtex ontology.

	Terminology	Assertions
Facts	A book is an entry A novel is a book has_author is a kind of human-creator has_editor is a kind of human-creator	*A Tale of Two Cities* is a novel *A Tale of Two Cities* has the author “Charles Dickens” *A Tale of Two Cities* has the year “1859”

Axioms	A book has a minimum of one human-creator A book has a minimum of one title A book has a minimum of one publisher A book has a minimum of one year	

**Table 2:** Ontology statistics.

	HistOnto	SemQ
Classes	24	20
Properties	25	38
Individuals	73	145

**Table 3:** Number of MCQ items generated.

Ontology	Individual-based	Transitive properties	Class-based	Total (filtered)
HistOnto	212	26	75	313 (235)
SemQ	796	69	145	1010 (798)

**Table 4:** MCQ design rules.

Rule	Description
General (G)	(R1) has a plausible distracter (R2) avoids excessive verbiage (R3) contains no negative or other counterintuitive wordings without underlining or special emphasis

Stem (S)	(R4) deals with a central problem (R5) has the to-be-completed phrase at the end

Responses (R)	(R6) are grammatically consistent with the stem (R7) do not necessarily repeat language from the stem (R8) are all of approximately the same length (R9) avoid the use of “all of the above” or “none of the above.” (R10) contain one correct answer

**Table 5:** Results of the MCQ generation task.

Metric	SemQ ontology	HistOnto ontology
Total number of worthy items	49 (82%)	36 (60%)
Average quality measure per MCQ item	8.8	8.8
Modification measure	Mode = 3	Mode = 0

**Table 6:** Reasons for MCQ major modifications.

Reason	Number of MCQ items
Distracters should be replaced (too easy)	6
MCQ item has less than four options	6
Some options are incorrect or invalid	4
No correct answer found within the options	2
Rephrasing of stem required	2
Options contain more than one correct answer	1
